# Excess Baggage for Birds: Inappropriate Placement of Tags on Gannets Changes Flight Patterns

**DOI:** 10.1371/journal.pone.0092657

**Published:** 2014-03-26

**Authors:** Sylvie P. Vandenabeele, Edward Grundy, Michael I. Friswell, Adam Grogan, Stephen C. Votier, Rory P. Wilson

**Affiliations:** 1 Biosciences department, College of Science, Swansea University, Singleton Park, Swansea, United Kingdom; 2 College of Engineering, Swansea University, Singleton Park, Swansea, United Kingdom; 3 RSPCA Wildlife department, Wilberforce Way, Southwater, West Sussex, United Kingdom; 4 Environment & Sustainability Institute, University of Exeter, Cornwall Campus, Penryn, Cornwall, United Kingdom; University of Zurich, Switzerland

## Abstract

Devices attached to flying birds can hugely enhance our understanding of their behavioural ecology for periods when they cannot be observed directly. For this, scientists routinely attach units to either birds' backs or their tails. However, inappropriate payload distribution is critical in aircraft and, since birds and planes are subject to the same laws of physics during flight, we considered aircraft aerodynamic constraints to explain flight patterns displayed by northern gannets *Sula bassana* equipped with (small *ca*. 14 g) tail- and back-mounted accelerometers and (larger *ca*. 30 g) tail-mounted GPS units. Tail-mounted GPS-fitted birds showed significantly higher cumulative numbers of flap-glide cycles and a higher pitch angle of the tail than accelerometer-equipped birds, indicating problems with balancing inappropriately placed weights with knock-on consequences relating to energy expenditure. These problems can be addressed by carefully choosing where to place tags on birds according to the mass of the tags and the lifestyle of the subject species.

## Introduction

Despite the unquestionable progress in our understanding of wild bird movements through the miniaturization of remote-sensing devices, the extra mass that these devices represent for their carriers has been cause for concern [1], [2]. In an attempt to overcome such device effects, Kenward [3] suggested that birds should not be fitted with devices representing more than 3% of their body mass. Despite the fact that this rule is an important first step as to reduce tagging impact, a recent study modelling bird flight indicated this was an over-simplification since other factors such as device-induced drag can influence the degree to which tags may impact their carriers [4]. Besides, major differences in morphologies, wing loadings and life-histories between bird species suggest that device mass effects should perhaps best be considered at a family or group level [4]. Undoubtedly such studies have helped refine our thinking with respect to how extra mass might impact flying birds but it does not address the important issue of device placement. Common sense would dictate, in agreement with basic laws of physics about stability in flight [5], that tags should be placed as closely as possible to the centre of gravity to minimise the potential for destabilization [6], [7]. However, this solution is somewhat at odds with suggestions made for diving birds, for example, where the explicit recommendation has been to place tags towards the rear of the bird to minimize drag (cf. [8]). Indeed, such arguments would appear particularly germane in the extreme case of plunge-diving birds, such as gannets (Sulidae), where the force applied to the tag as the bird enters the water is likely to be extreme [9], [10]. Not only will such force presumably impact the bird but also makes device attachment problematic. One technique that seems to have solved, or at least reduced, this plunging impact problem is the practice of fixing tags to the underside of the tail using tape (e.g. [11], [12]). This appears to result in the tag being protected by the feet during the plunge and has resulted in tags being attached for periods extending from days to weeks (e.g. [11], [13]). The effect of this deviation from the bird's centre of gravity, however, is unknown.

In aircraft, the weight distribution has to be carefully managed to ensure the position of the centre of gravity is within specified limits. Since a significant proportion of the aircraft weight is payload (cargo and/or passengers), the weight balance has to be calculated for every flight and adjusted by moving the location of payload as necessary [14]. For long-haul flights the weight of fuel is also significant and distribution amongst the multiple on-board fuel tanks may be adjusted to ensure the weight is balanced [15]. There are two main reasons why the centre of gravity is controlled so carefully in aircraft. First, in straight and level flight the aircraft is *trimmed* so that the aerodynamic forces, moments and weight are in equilibrium. In practice, the angle of the elevator and/or horizontal tail is adjusted to maintain altitude; if the centre of gravity is outside the specified limits then either the tail is unable to generate sufficient force to maintain equilibrium, or the drag penalty is too high. Secondly, moving the centre of gravity aft reduces the longitudinal static stability of the aircraft [16]. This makes the aircraft more responsive but also more difficult to fly. These considerations of weight balance in aircraft should be just as applicable to birds and so we used it as a framework to investigate the effects of tags, and therefore payload mass and position in birds.

For this, we attached accelerometers to northern gannets (*Sula bassana*), a species that habitually plunge-dives to capture prey [9], [10], and which has been subject to an appreciable number of tagging studies using both back- and tail-mounted devices (e.g. [13], [17], [18]). Since accelerometers can give information on both body posture [19], [20] and the energy invested in movement [21]–[23], we sought to define a protocol to identify the extent to which back- and tail-mounted tags may differentially affect birds with a view to minimizing potential device effects. Our point of departure is that non-centrally mounted payloads will affect flight capacity in these birds in the same manner that it does in aircraft since the principles of flight in both birds and planes are comparable [24].

## Methods

### Study site and device deployment

**Figure 1:**
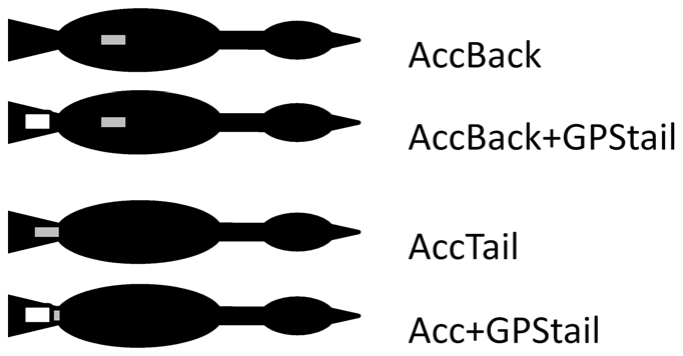
Dorsal placements of external devices fitted to gannets. Schematic representation of a gannet wearing devices at different positions as to test the effect of device mass and placement on flying behaviour and energetics. NB All devices placed on the tail were attached to the underside of 2 to 3 central feathers using Tesa tape.

The study was conducted during July-August 2011 and 2012 at the breeding colony of northern gannets located on Grassholm, Wales, UK (51°43′N, 05°28′W). The second field trip in 2012 was mainly to try increase the sample size but since the priority was given to another study conducted at the same time on the same colony, very limited number of additional birds could be added. Permission to access Grassholm Island was provided by the Countryside Commission for Wales (now called Natural Resources Wales) and the Royal Society for the Protection of Birds (the landowner). The handling of the birds and attachment of unconventional marks was carried out under licence from the British Trust for Ornithology. A total of 19 chick-rearing gannets were caught on the nest at change-over and equipped with data-loggers attached to the feathers using waterproof Tesa tape [25], [26]. No measurements including that of body mass and other morphometric were taken in order to reduce handling time and therefore avoid overstressing the birds. Neither blood samples were taken for the same reason. Fourteen birds were fitted with a tri-axial accelerometer on the back (X6-2mini accelerometers, 8.5 cm L*2.4 cm W*1.6 cm D including the waterproof case, Gulf Coast Data Concepts LLC, Waveland, US) and of these, 5 had a dummy GPS tag (i-gotU GPS Travel Logger GT-600 (Maplin Electronics Ltd, 6.2 cm L*6.2 cm W*1.7 cm D, *ca*.30 g, which is used widely in this species) on the back, 4 with a dummy GPS on the tail and 5 with no further device (Figure 1, Table 1). In addition, to look at detailed tail posture and movements, 6 birds were fitted with an accelerometer under the tail with, and without the presence of a GPS tag (Figure 1, Table 1). Accelerometers consisted of a circuit board and battery that had been removed from their original housing and coated with epoxy-resin. Once programmed via USB connection and just prior to deployment, they were sealed in a waterproof heat-shrink tubing package. The whole system (waterproof case included) weighed between 13 and 15 g. With the addition of a dummy GPS weighing between 28 and 30 g, birds carried a total mass ranging from 13 g (accelerometer alone) to 45 g (accelerometer plus dummy GPS) accounting for 0.4 to 1.5% of the adult body mass (*ca*. 3 kg) [9], [27], depending on the type of devices deployed (Table 1). The lateral compression and positioning of all attached devices were assumed to have minimal impact on the bird's streamlining. The placement of the device on the back was carefully chosen based on the results of a wind-tunnel study, which looked at the effect of tag position on drag [28]. More precisely, the device was placed as close as possible to the centre of gravity. The acceleration of the bird was recorded continuously at a sampling rate of 40 Hz in each of the 3 main orthogonal axes (dorso–ventral [heave axis], anterior–posterior [surge axis] and lateral [sway axis]) with 16-bit resolution for the duration of at least one foraging trip. When back on the nest, the birds were recaptured and the equipment removed.

**Table 1:** Details of the device deployment conducted on northern gannets (*Sula bassana*).

Groups	Sub-groups	Number of birds	Device deployed	Position	Device mass
Non-GPS birds	AccBack	4	Accelerometer	Middle back	13–15 g
	AccTail	3	Accelerometer	Tail	13–15 g
GPS birds	AccBack+GPStail	4	Accelerometer	Middle back	13–15 g
			+ Dummy GPS	Tail	28–30 g
	Acc+GPStail	3	Accelerometer	Tail	43–45 g
			+ Dummy GPS	Tail	
Acc+GPSback (excluded after deployment failure)	Acc+GPSback (excluded after deployment failure)	5	Accelerometer	Middle back	13–15 g
			+ Dummy GPS	Middle back	28–30 g

Devices were deployed on adult (chick-rearing) gannets (*Sula bassana*) to examine the potential effects of the position of extra mass on their flying behaviour. Of the 14 birds equipped with an accelerometer on the back, 8 (4 in the ‘AccBack’ group and 4 in the ‘AccBack+GPStail’ group) were included in the analysis. Another 6 birds were included in the analysis which had been fitted with accelerometers on their tail, with, and without, GPS (‘Acc+GPStail’ and ‘AccTail’ group respectively) (See Figure 1).

### Data analysis

**Figure 2:**
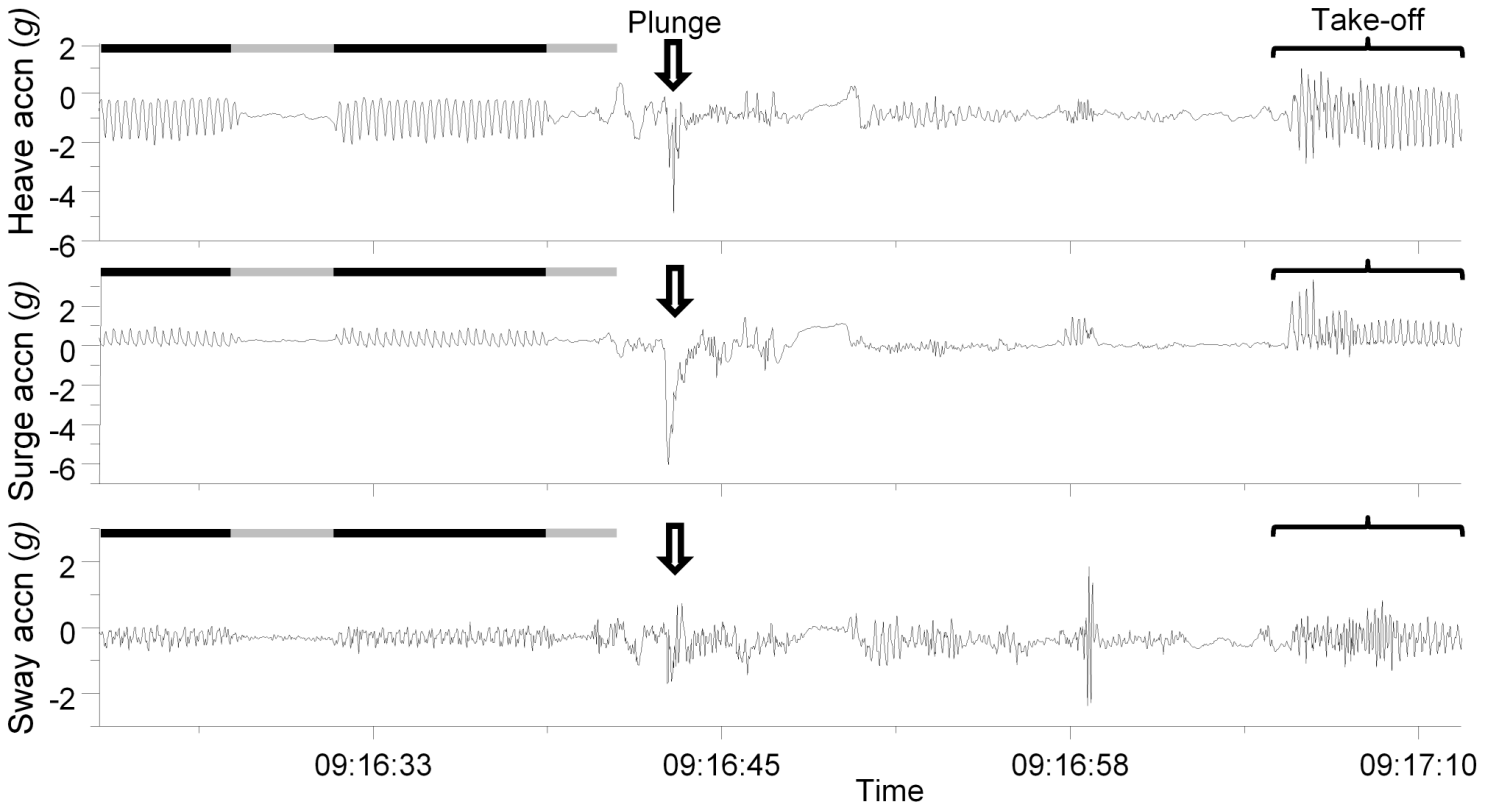
Tri-acceleration signal showing the behaviour of a gannet during a foraging trip. Example of gannet behaviour recorded by a data-logger (X6-2mini accelerometers, GCDC LLC, Waveland, US) showing the tri-axial acceleration signature during flight (flapping – black horizontal bars and gliding - grey bar) just before and after a plunge dive (indicated by the arrow followed by phase on sea surface before flapping to take-off).

After recapture, the devices were retrieved and data downloaded for later analysis using Origin (version 8.5.1, OriginLab Corp., USA) and Excel (version 2010, Microsoft inc., USA) software. Different behaviours could be identified based on the frequency and amplitude of the accelerometry signal in the three axes (Figure 2). First, the foraging trip duration was determined based on the acceleration signal which allowed clear identification of take-off and landing (e.g. Figure 2 for take-off). Then, the analysis focused on periods of 10 minutes of regular flight extracted between 30 minutes to an hour after departure from the breeding colony. One period of 10 min flight was randomly selected for each bird. This period was chosen because the birds had an empty gut, being caught on change-over meant that birds had conducted long periods of chick-rearing and foraging does not normally occur until a considerable distance from the colony at least for the gannet colony on Grassholm island for which a foraging range of hundreds of kilometres has been calculated [29]. This was to reduce the confounding effects of a food load on flying behaviour. An approximation of the static (gravity-based) acceleration was derived from the raw acceleration recorded by the loggers using a running mean over 2 s (cf. [30]). Simple trigonometry was used to derive the pitch angle of the bird during flight using the acceleration data recorded in the anterio–posterior axis (cf. [31]) after correcting for possible variance in attachment angle by using the angle when the gannet was resting on the sea surface as zero. The calculation of this pitch angle was done using the acceleration data collected from the devices attached on the back of the birds to assess body posture (i.e. body pitch angle), as well as those placed under the tail (i.e. pitch of the tail) to examine tail posture. Once the pitch angle of each bird was determined, the data of those which belonged to the same experimental group were pooled before comparing the groups (i.e. non-GPS birds vs. GPS birds, see Table 1 for details on the groups) using a Mann-Whitney U test.

The acceleration data collected on the back as well as on the tail of the birds was subjected to further analysis to determine flight energetics. For this, we derived a proxy for movement-based energy expenditure termed the Overall Body Dynamic Acceleration (ODBA) [21]. To calculate this metric, the static acceleration for each of the orthogonal acceleration axes was first subtracted from the relevant raw acceleration values to obtain the dynamic component of acceleration. The absolute values of these dynamic acceleration data were then summed over the 3 channels to obtain the ODBA [20], [21]. ODBA was determined for the same periods of 10 minute flight previously considered.

### Running variance to identify behavioural signatures

During flight, flapping and gliding exhibit distinct acceleration profiles in both the surge and heave axis (Figure 2). We used a running variance of the surge acceleration so as to identify flapping from gliding phases. The running variance is the average residual which was calculated over periods of one second according to:
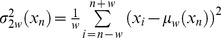
(1)where *w* is the time window considered, *x* is the data value at index *n* and 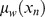 is the mean value over the same time window.

**Figure 3:**
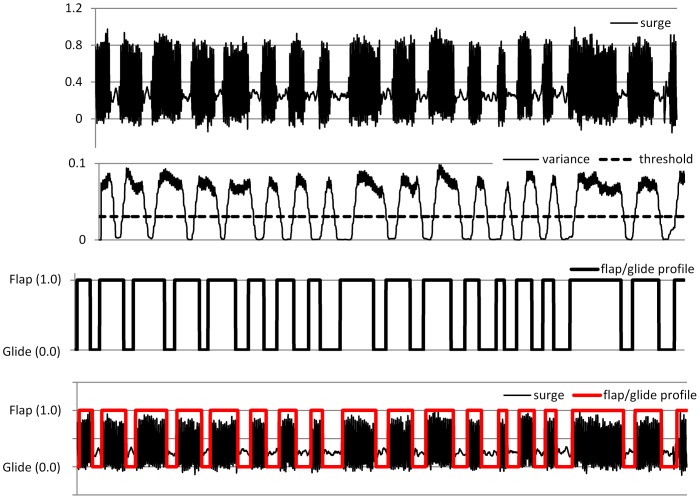
Identification of the different phases during the flight of a gannet. Flapping and gliding behaviours recorded in the surge (antero-posterior) acceleration axis by an accelerometer fitted to the back of a northern gannet (*Sula bassana*) (top) are separated by the running variance (middle-top), producing a binary signal (middle-bottom). The alignment of this signal with the raw data supports the behavioural classification (for details see text).

Taking the average variance across the whole 10 min flight period (*σ(X)*) and using it to compute a binary classification gives an accurate signal representing the 2 flight states:
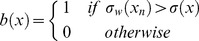
(2)where a value of 1 represents flapping behaviour and 0 represents gliding behaviour (or, specifically, not flapping, which we assume to be gliding as the animal was in flight for the whole period) (Figure 3). Based on this binary classification of the flight data, it was then possible to describe and compare profiles of flapping and gliding behaviours among the birds.

## Results

All 19 birds returned to their nest. Four of the five birds in the ‘Acc+GPSback’ treatment group (Table 1) lost their tags and therefore were excluded from analysis. Consequently, data were collected from 14 birds of which, four with accelerometers on the back (‘AccBack’) and four with accelerometers on the back plus GPS units under the tail (‘AccBack+GPStail’), were analysed (Table 1). Six other birds were included in the analysis which carried accelerometers on their tail with, or without, GPS (‘AccTail’ and ‘Acc+GPStail’ respectively; see Table 1). AccBack plus AccTail birds formed the group thereafter referred as non-GPS birds and the AccBack+GPStail with the Acc+GPStail formed the group referred as the GPS birds. The entire analysis was performed considering these two groups. However, when examining specifically at the pitch angle or the energetics via the proxy ODBA, the distinction was made between the birds carrying the accelerometer on the back or under the tail since the signal obtained from these two locations was different and not comparable.

**Figure 4:**
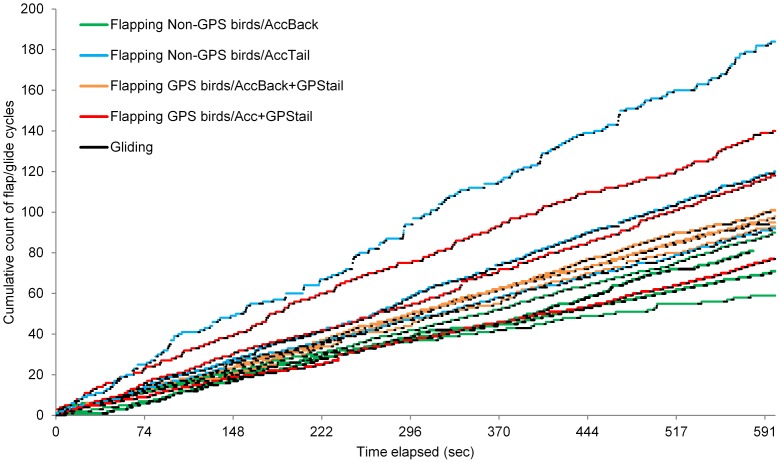
Details on the cyclic pattern during the flight of gannets. Cumulative count of flap/glide cycles over time measured for 14 gannets during a 10 minute flight after their departure from the breeding colony. The birds were equipped with miniature tri-axial accelerometers on their back or tail without (AccBack and AccTail respectively) or with, a dummy-GPS on their tail (AccBack-GPStail and Acc+GPStail respectively). The duration of the flapping period (in green, blue, orange or red depending on the bird group) is also shown relative to the duration of the gliding period (in black for all groups of birds).

The duration of foraging trips ranged from 7 to 28 hours being significantly shorter for birds with GPS compared to those with accelerometers only (Mann-Whitney U test, *z* = −2.33, *P* = 0.02; mean ± sd  = 25±2 h for non-GPS birds and 15±9 h for GPS birds). Previous studies conducted on gannet colonies nesting around the UK have reported foraging trips of non-equipped birds to be around 24 h [32], [33] which is closer to what was observed for non-GPS birds. The running variance analysis was performed on the surge acceleration signal allowing the identification and comparison of flapping and gliding behaviours between the birds. No clear difference was found in the amount of time spent flapping *per se* or relative to gliding (Mann-Whitney U test, *P*>0.05; see Table 2). However and interestingly, it seems like there was more variation in the flap/glide cyclic pattern for the GPS birds as well as for the birds with the accelerometer under the tail compared to those with an accelerometer on the back. Apart from one bird fitted with both devices under the tail which flap/gild profile appeared similar to that of the AccBack birds (all four green profiles and one red profile, Figure 4), all the other birds (blue, orange and red profiles, Figure 4) executed more transitions between the two states resulting in a larger number of flap/glide cycles over the 10 min flight period considered.

**Table 2:** Statistics for the flying behaviour of northern gannets (*Sula bassana*).

	Non-GPS birds		Non-GPS birds	
	AccBack	AccTail	AccBack+GPStail	Acc+GPStail
**Foraging trip duration (h)**	26/25/26/26	28/25/21	7.5/7/24/10	26/7/24
**Total number of flap/glide cycles**	90/75/65/89	97/191/131	98/93/97/106	83/150/126
**Flap/glide duration ratio**	1.61/3.19/3.38/1.55	1.45/4.31/1.28	2.17/4.86/2.26/1.52	2.23/4.22/1.21
**Total time flapping duration**	352/440/443/352	353/487/340	392/475/400/345	418/489/331
**ODBA(mean ± SD)**	0.62±0.46	0.64±0.47	0.68±0.62	0.57±0.51

Statistics calculated for all the 14 adult gannets using data from a 10 minute flight period.

The birds were equipped with a miniature tri-axial accelerometer on their back or tail and with, or without, a dummy-GPS underneath their tail ((Non-GPS birds and GPS birds respectively). Each column displays the data for all the birds of each group, between 3 or 4 individuals.

**Figure 5:**
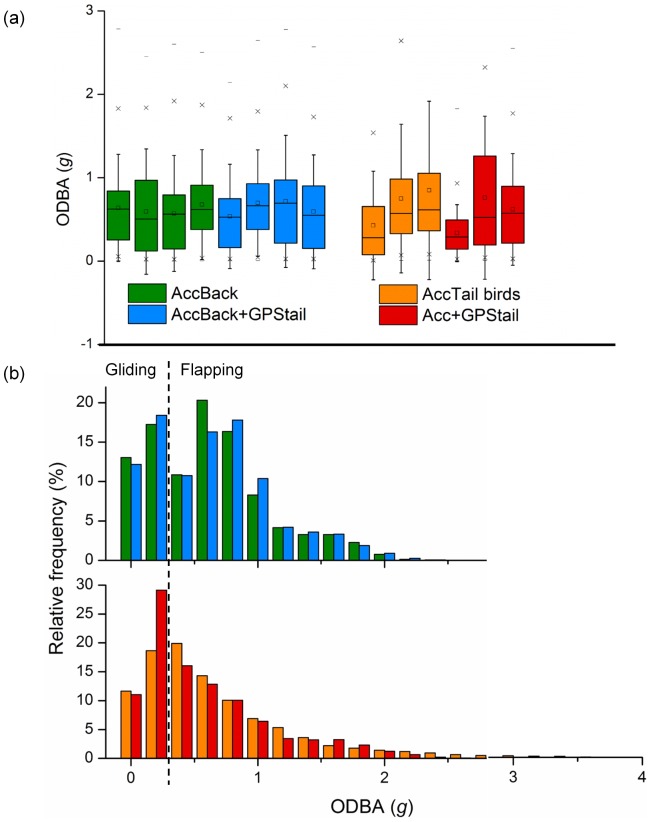
Comparison of the energy expenditure of flying gannets with or without GPS. (a) Box charts of the proxy for energy expenditure ODBA [21] calculated for flying gannets fitted with solely an accelerometer on their back or tail (green and blue bars respectively grouped as the non-GPS birds), or with, in addition to the accelerometer, a GPS under the tail (orange and red bars respectively grouped as the GPS birds). This illustrates that the mean ODBA remains similar between the groups despite their different treatments. (b) Frequency distribution of ODBA obtained for the different groups of birds cited above. This figure shows that a higher number of ODBA values is obtained for GPS birds compared to non-GPS birds.

As mentioned before, the acceleration signal and consequently the derived proxy for energetics, ODBA, obtained from the back and tail locations were not comparable. Therefore, only comparisons between the non-GPS birds versus the GPS birds for the two scenarios (back and tail) separately could be achieved. More precisely, the comparisons were between the following sub-groups: AccBack vs. AccBack+GPStail birds and AccTail vs. Acc+GPStail birds. While the mean ODBA did not seem to differ much between the birds (Table 2, Figure 5a), the frequency distribution even if broadly similar yet appeared significantly different (Mann-Whitney U test, *z* = 7.52, *P*<0.001 and *z* = 30.56, *P*<0.001 respectively for the back and tail scenario case, Figure 5b). One noticeable difference was that GPS birds presented a greater proportion of high ODBA values during the gliding phase mainly (i.e. ODBA <0.4 *g*) than non-GPS birds (Figure 5b). This effect was even more accentuated for birds fitted with both an accelerometer and GPS under the tail (Acc+GPStail birds in red, Figure 5b) compared to birds with just an accelerometer under the tail (AccTail in ornage, Figure 5b). However, it cannot be excluded that this significant difference may be due to the power of the non-parametric test performed on a large dataset.

Unlike for other species [23], no calibration is yet available between the proxy for energy expenditure ODBA and the metabolic rate of gannets. Thus, the ODBA could not be converted into values of energy consumption spent per unit of time. Instead, an estimation of the amount of energy expended by flying gannets with and without a 30 g payload was calculated using the free access software Flight developed by Prof. Pennycuick (Flight 1.24 software accessible at http://www.bristol.ac.uk/biology/people/colin-j-pennycuick/index.html, cf. [24]. An increase of 2.4% was found with the unequipped bird having to expend 12.6 J/s in comparison to the 12.9 J/s expend by a bird carrying a 30 g backpack.

**Figure 6:**
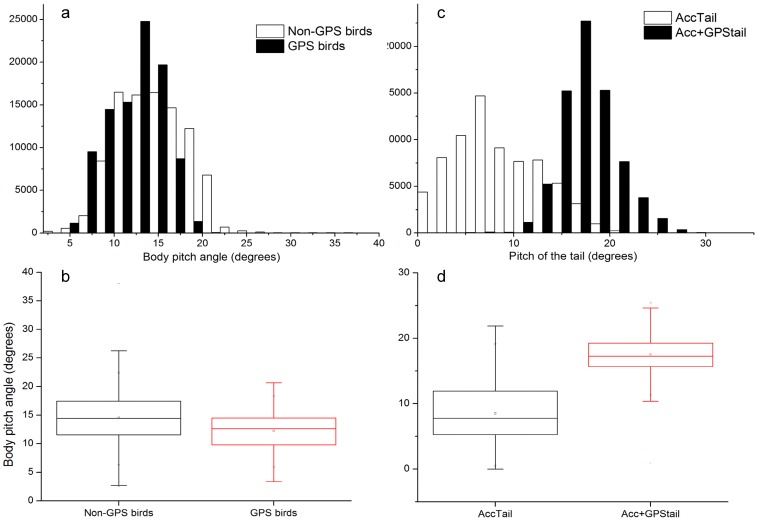
Differences in the flight angle of gannets with or without GPS. Frequency distributions (a, c) and box plots (b, d) of the body pitch angle of gannets (see text for full explanation) calculated over 10 minute flight period and while carrying different payloads at different positions. (a, b) Non-GPS birds (N = 4) were fitted with 15 g accelerometers on their back as were GPS birds (N = 4) which, in addition, carried a 30 g dummy GPS also under the tail. No significant difference in the body pitch angle was revealed between the 2 groups. (c, d) AccTail birds (N = 3) were fitted with 15 g accelerometers under their tail as were Acc+GPStail birds (N = 3) which, in addition, carried a 30 g dummy GPS also under the tail. The difference in the tail pitching moment between the 2 groups is significant (Mann-Whitney U test, *z* = 294.9, *P*<0.001).

The flight pitch angle of the birds did not obviously change between the experimental groups (Mann-Whitney U test, *P*>0.05; see Figure 6a and 6b) but was significantly different at the tail level with the birds carrying the heaviest payload (accelerometer + GPS) showing a higher pitch than the other birds carrying just an accelerometer under the tail (Mann-Whitney U test, *z* = 294.9, *P*<0.001; Figure 6c and 6d).

## Discussion

To be able to study animals both in an ethically and scientifically correct way using external tags can be a real challenge. This appears particularly true for birds which behaviour and ecology can significantly be affected by the presence of devices [34]. In addition to the common issues related to tag size and mass, position also can be critical. This study considered the middle back and the tail as two common locations for attaching tags to free-living gannets. The addition of tags at different positions on a bird has two major aeronautical effects, aerodynamic and inertial. The middle back location will increase the drag of the bird slightly (and hence the energy requirements) but should not significantly affect the lift, which is generated mainly by the wings [35]. The tail location has more potential to disrupt the flow depending on the size of the device relative to the size of the tail. The location under the tail will effectively reduce the camber of the tail and, in aeronautical terms, should reduce the lift from the tail.

The inertial properties of our study gannets were changed by the addition of GPS loggers in two significant ways. The increased mass means that the GPS birds have to generate more lift to fly, and hence use more energy through increased induced drag [24], [35]. The second effect is to move the centre of gravity rearwards, and hence the trim of the bird (when the total forces and moments are zero) will have to change. The middle back location will be approximately above the centre of gravity and thus will have little effect on the trim. The mass of the device at the tail location will cause a positive pitching moment with a corresponding increase in the pitch angle, and therefore an increase in height and a decrease in speed that would have to be matched through an increase of lift from the tail. Given also that the device aerodynamics will give a decrease in lift from the tail, this means that the angle of attack of the tail would have to be increased significantly, resulting in increased drag from the tail. These effects are expected to be greater if the tag is placed on top of the tail instead on underneath mainly because of the airflow being further disrupted therefore creating more drag.

Despite a limited dataset, the results support the predictions that, first, additional weight can affect the flight patterns of flying birds, even though we used no tag system that exceeded the 3% limit proposed by Kenward ([3], but see [4]). First, GPS birds were reported to spend less time at sea than accelerometer-equipped birds. This is probably due to an increase in energy requirements, as shown by the difference in ODBA mainly during the gliding phase, which even if relatively small, may not be sustainable over time and forced the equipped birds to come back earlier to the colony to rest. That is what the calculations made using the program Flight seem to indicate. What may seem to be very little difference (i.e. 12.6 J/s vs. 12.9 J/s for an unequipped bird vs. a bird with a GPS) can in reality, when considered at the scale of the entire foraging trip, have a more significant impact. Moreover, these estimations are probably an underestimation of the real difference between unequipped birds and GPS birds mainly because the calculations do not take into account the placement of the package. Indeed, the software assumes that the payload is placed in the middle of the back close to the centre of gravity and with, therefore, no or little effect on the balance and flight angle.

Interestingly, and despite the fact that foraging trip duration is considered to be a good indicator of device effects, the effect found in our study appears to be different to what was reported in previous studies. It has been observed in some occurrences that equipped birds extended their foraging trips compared to unequipped birds [36], [37], [38], [39]. It is, however, important to note that most of these studies were performed on penguins. In other cases, no change in the length of foraging trips could be detected between equipped and control birds [40], [41], [42]. Therefore, such discrepancy in the observations made about the foraging trip length of equipped birds could only indicate that, different species respond differently to the attachment of tags. Furthermore, it shows that tag effect is a complex issue which should not be investigated based on the assessment of a single parameter such as foraging trip length.

The critical point that our study adds to all the considerations about how extra weight may affect flying birds is that placement of this weight may play a key role with regard to how much impact it will cause. That statement stems from a couple of observations starting with the fact that the birds which showed the most disparity in their flap/glide pattern with a higher number of transitions between flapping and gliding phases were those fitted with a device under their tail, whether it was a light accelerometer or a heavier GPS unit. Another argument in this favour is that the amount of energy spent during the gliding phase seems most expensive for birds with the most extra weight under their tail. Therefore, the combination between how much weight is fitted to the bird and where this weight is placed probably is a better predictor of how much the equipment will affect birds in terms of their flying behaviour and energetics.

One non-exclusive explanation why the placement of devices under the tail may have a greater impact than devices placed closer to the centre of gravity relates to the observed change in the pitch of their tail. This supports the prediction that birds with tail-located devices would undergo a change in their flying posture as to adjust their trim (see above). Since the birds were not used to the devices, they may initially set their tails to the expected angle, which would cause a pitch-up moment with a corresponding increase in height and decrease in speed. An appropriate response to this would be to start flapping to prevent stalling which would explain why birds fitted with a device under their tail presented a higher number of flap/glide cycles. Similarly, initiation of flapping after shorter gliding phase could be linked to an increase in the sink rate during gliding resulting from the attached tags. Calculations using the Flight software indicates that GPS-equipped northern gannets should experience an increase of 6.3% in their sink rate (being 0.64 m/s compared to 0.60 m/s for GPS birds and non-GPS birds, respectively). Descending the air column in a faster way and to avoid reaching too low altitudes, equipped birds have to start flapping after only short periods of gliding.

**Figure 7:**
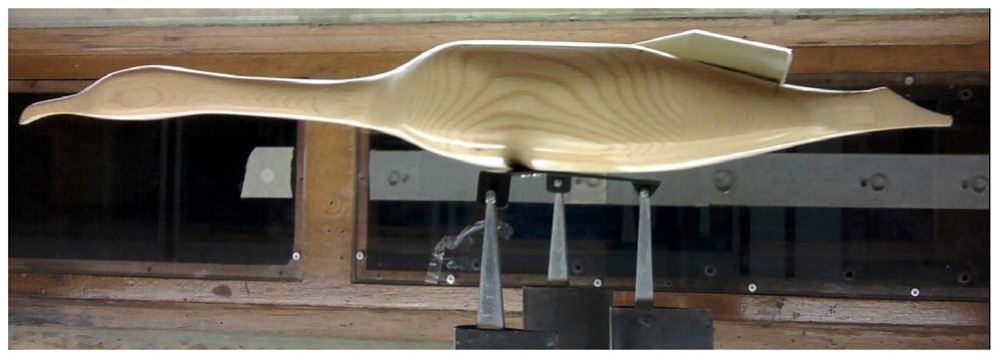
Photo of the suggested ideal placement for attaching tags on diving birds which also spend a significant amount of time flying. This is a bird model fitted with a dummy tag placed in a wind tunnel to measure the drag associated with various tag shapes and placements. The dummy tag shown on this picture is placed at the exact position for which the drag measured was minimum in air and water conditions (unpublished data).

Equipment of northern gannets with such tail-mounted packages may compromise their capacity to travel efficiently the long distances they cover during foraging [18], [43] with possible knock-on effects relating to their capacity to exploit highly variable prey abundance and distribution [11], [44]. Thus, the case of how to equip plunge-diving birds would appear problematic, with back-mounted tags increasing drag, especially during the plunge (where deceleration can be up to 6 *g*; Figure 2 middle panel with surge acceleration), and being subject to device loss as a result, while tail-mounted units likely upset both the trim of the bird and the tail angle with all the problems that these engender. This highlights the challenge that it represents to attach tags to species living at the interface of two environments as different as air and water which means that both mass and drag are critical if to minimise the impact of the tag on the carriers. Based on these results in combination with wind tunnel data of drag measured on a bird model wearing devices at different positions [28], we would recommend to attach tags to the space in between the middle back and the lower back (Figure 7). This is to ensure that the added mass of the device is kept relatively close to the centre of gravity which is important when flying while minimising the increase in the frontal cross-sectional area of the bird as to avoid any substantial increase in drag when swimming underwater. This is valid for any bird species known to be flying a significant amount of time while still relying on diving to forage. For species which do not fly like penguins, the lower back is certainly the best position where to attach tags as device mass is less of an issue in that particular case. At the other end of the spectrum, for strictly flying birds, the middle back placement may still represent the best option to attach tags with ideally a streamlined shape as to avoid airflow disruption.

Perhaps the best way forward is to work on centrally mounted tags with minimum drag and enhanced stability [45], [46], which can be designed using Computational Fluid Dynamics and Computer Aided Design [unpublished data] in combination with more robust attachment procedures such as bird-friendly Silastic harnesses [47] which hold units in place more securely than do simple tag/feather attachment systems [48]. Either way, it is clear that we should not continue attaching tags to birds without giving the consequences of tag placement more thought.
